# Primary blast injury-induced lesions in the retina of adult rats

**DOI:** 10.1186/1742-2094-10-79

**Published:** 2013-07-02

**Authors:** Ying-Ying Zou, Enci Mary Kan, Jia Lu, Kian Chye Ng, Mui Hong Tan, Linli Yao, Eng-Ang Ling

**Affiliations:** 1 Department of Pathology, Faculty of Basic Medical Sciences, Kunming Medical University, 1168 West Chunrong Road, Kunming, P R China 650500; 2 Department of Anatomy, Yong Loo Lin School of Medicine, National University of Singapore, 4 Medical Drive, Block MD10, Singapore 117597, Singapore; 3 Defense Medical and Environmental Research Institute, DSO National Laboratories, 27 Medical Drive, Singapore 117560, Singapore

**Keywords:** Blast, Retina, Nitric oxide synthase, Vascular endothelial growth factor, Aquaporin-4, Inflammation, Apoptosis

## Abstract

**Background:**

The effect of primary blast exposure on the brain is widely reported but its effects on the eye remains unclear. Here, we aim to examine the effects of primary blast exposure on the retina.

**Methods:**

Adult male Sprague–Dawley rats were exposed to primary blast high and low injury and sacrificed at 24 h, 72 h, and 2 weeks post injury. The retina was subjected to western analysis for vascular endothelial growth factor (VEGF), aquaporin-4 (AQP4), glutamine synthethase (GS), inducible nitric oxide synthase (NOS), endothelial NOS, neuronal NOS and nestin expression; ELISA analysis for cytokines and chemokines; and immunofluorescence for glial fibrillary acidic protein (GFAP)/VEGF, GFAP/AQP4, GFAP/nestin, GS/AQP4, lectin/iNOS, and TUNEL.

**Results:**

The retina showed a blast severity-dependent increase in VEGF, iNOS, eNOS, nNOS, and nestin expression with corresponding increases in inflammatory cytokines and chemokines. There was also increased AQP4 expression and retinal thickness after primary blast exposure that was severity-dependent. Finally, a significant increase in TUNEL+ and Caspase-3+ cells was observed. These changes were observed at 24 h post-injury and sustained up to 2 weeks post injury.

**Conclusions:**

Primary blast resulted in severity-dependent pathological changes in the retina, manifested by the increased expression of a variety of proteins involved in inflammation, edema, and apoptosis. These changes were observed immediately after blast exposure and sustained up to 2 weeks suggesting acute and chronic injury mechanisms. These changes were most obvious in the astrocytes and Müller cells and suggest important roles for these cells in retina pathophysiology after blast.

## Background

Recent reports showed that 64% of war injuries are caused by blasts [1,2]. Traumatic brain injury (TBI) is so prevalent that it has been described as the ‘signature wound of the war on terror’ [3], and has been the focus of many studies. As personal ballistic protection of the head and torso improves for soldiers, increasing numbers of injuries to unprotected areas such as the face and eyes may be expected [4]. Morley *et al.* reported that ocular injury is a frequent cause of morbidity in blast victims, affecting up to 28% of blast survivors. The most common blast eye injuries include corneal abrasions and foreign bodies, eyelid lacerations, open globe injuries, and intraocular foreign bodies. Injuries to the periorbital area can be an additional source of significant morbidity, and ocular blast injuries have the potential to result in severe vision loss. Although secondary blast injuries resulting from flying fragments and debris cause majority of eye injuries among blast victims [5], primary blast injury to the eye has also been documented, but the differentiation between air-blast and fragment etiologies in the reports is not clear [6]. There is limited conclusive evidence that primary ocular blast injury occurs in survivors of explosions. However, some case reports do surmise its occurrence and it cannot be unequivocally ruled out. For example, it has been suggested that higher overpressures produce more life-threatening ocular injuries. The development of enhanced blast weapons may result in an increased incidence [6]. The main focus on blast ocular injury has been to consolidate the clinical data for statistical review in humans [4,5,7]. However, these studies do not investigate the pathological changes and molecular mechanism of primary blast ocular injury. Very few studies have focused on primary blast injury to the eye [8] and reports on retinal changes following blast injury are lacking. It remains to be investigated whether the retina after exposure to primary blast would exhibit any pathological changes.

In light of the above, the aim of this study is to determine whether primary blast injury can induce retinal lesion and if it does occur, how the retinal neurons and glial cells would respond. According to previous studies, primary blast injury induced inflammation, apoptosis, edema in brain [9], spinal cord [10], and lung [11]. We therefore hypothesized that inflammation, edema, apoptosis can also occur in the retina after blast. We also hypothesized that the expression of markers that are evidently linked to retinal injuries such as nitric oxide synthase (NOS), vascular endothelial growth factor (VEGF), aquaporin-4 (AQP4), nestin, glutamate, and glutamine synthetase (GS) would be altered following primary blast injury. These molecules have been reported to be altered in the retina exposed to hypoxia [12] and smoke inhalation [13]. Furthermore, VEGF [14-16], AQP4 [17-19], NOS [20-22], nestin [19,23], GS [19], and glutamate [24], are known to be associated with vasodilation, tissue edema, and inflammatory reaction which have all been reported to be involved in retinal injury pathophysiology. We therefore sought to determine if these factors would be altered in the adult rat retina after blast exposure.

## Methods

### Animals

Adult male Sprague–Dawley rats (*n*=129) (300–350 g) were used for the primary blast injury model in this study. All rats were housed under conditions of room temperature on a 12-h regular light/dark cycle with *ad libitum* access to food and water. All animal experimentation protocols in this research project were approved by the DSO Institutional Animal Care and Use Committee (protocol number DSO/IACUC/09/74). All efforts were taken to minimize the number of rats used and their suffering. Table 1 shows the number of animals used for blast exposure and for the various tests investigated.

**Table 1:** Number of rats used in different experiments for the whole study

	**Sham (sham)**	**BH 24 h**	**BL 24 h**	**BH 72 h**	**BL 72 h**	**BH 2 w**	**BL 2 w**	**Total**
Inflammatory cytokines assay	3					3	3	9
Western blotting analysis	4	4	4	4	4	4	4	28
Glutamate assay	3	3	3	3	3	3	3	21
Double immunofluorescence	4	4	4	4	4	4	4	28
Nitrite assay	4	4	4	4	4	4	4	28
Detection of apoptosis	3			3	3	3	3	15
Total								129

### Blast exposure

A total of 5 kg of 2,4,6-trinitrotoluene (TNT) with a penta-erythritol tetra-nitrate (PETN) booster was detonated at a height of 1 m. Four metal cages were set up at 1 m height and at 2 m and 3 m alternately from the blast source with a pressure transducer next to each cage. To avoid secondary blast injuries, body armor (BA, customized acrylic casing) was secured to the cage with cable ties and the metal cage shut tight with cable ties. All the animals were randomly grouped into: (1) Sham (sham), where the subjects were not exposed to blast but anesthetized; (2) Blast high (BH) where subjects were exposed to a single blast at approximately 480 kPa blast overpressure (BOP) at 2 m from blast source; (3) Blast low (BL) where subjects were exposed to a single blast at approximately 180 kPa BOP at 3 m from blast source. Up to eight animals were strapped loosely using Velcro to a metal mesh cage at the specified distances while animals in BA only had their head exposed in a forward-facing position. The head was prevented from moving by placing a sponge around the head at the openings of the body armor. Exposed areas of the animal were covered in ultrasound gel to minimize dehydration and singeing of fur. Animal subjects (both sham and blast subjects) were anesthetized prior to blast exposure with intraperitoneal injections of ketamine (75 mg/kg) and xylazine (10 mg/kg). Anesthetized sham animals were kept in cages away from the blast site. Immediately after blast exposure, the animals were assessed for any gross or penetrating facial and body injuries. The animals were euthanized at 24 h, 72 h, and 2 weeks post blast for the various tests investigated.

### Inflammatory cytokines and chemokines assay

The relative concentrations of 12 proinflammatory cytokines in the retina lysate of sham rats and those subjected to primary blast injury (*n* = 3 each at 24 h, 72 h and 2 weeks; BH and BL) were determined with a Rat Inflammatory Cytokines Multi-Analyte ELISArray kit (Qiagen, CA, USA, catalogue number Mer004A). The tissue homogenates for the ELISArray measurements were prepared as for western blotting and ELISArray measurements were performed according to the manufacturer’s protocol.

### Western blotting analysis

At designated time-points post blast exposure, the animals were anesthetized with ketamine (75 mg/kg) and xylazine (10 mg/kg) intraperitoneally and sacrificed with cardiac puncture. After sacrifice, fresh retinal tissue from sham rats (*n* = 4) and blast exposure rats (*n* = 4 each at 24 h, 72 h, and 2 weeks post BH and BL exposure) were removed, snap-frozen in liquid nitrogen, and stored at −80°C. The retina tissue proteins were extracted using a protein extraction kit (Pierce Biotechnology, Inc., Rockford, IL, USA) containing protease inhibitors. All procedures were carried out at 4°C. Homogenates were centrifuged at 15,000 × g for 15 min and the supernatant collected. Protein concentration of samples was determined by the Bradford method using bovine serum albumin (Bio-Rad Laboratories, Hercules, CA, USA). Samples of supernatants containing 25 μg of protein were heated to 95°C for 5 min and were separated on 8% sodium dodecyl sulphate-polyacrylamide gels (for iNOS, nNOS, eNOS, and nestin) and 12% sodium dodecyl sulphate–polyacrylamide gels (for GS, VEGF, and AQP4) using a Mini Protean II apparatus (Bio-Rad Laboratories). Protein bands were electroblotted onto nitrocellulose membranes (Bio-Rad Laboratories) using a semi-dry electrophoretic transfer cell. The membranes were washed with Tris-buffered saline (TBS)-0.1% Tween buffer and then blocked with 5% w/v non-fat dry skim milk for 1 h at room temperature. After this, they were incubated with primary antibodies (Table 2) in blocking solution overnight on a shaker at 4°C. After rinsing with TBS-0.1% Tween, the membranes were incubated with horseradish peroxidase-conjugated secondary antibody (1:2,000-10,000; Pierce Biotechnology, Inc.) for 1 h. Proteins were revealed by an enhanced chemiluminescence detection system according to the manufacturer’s instruction (Super Signal West Pico Horseradish Peroxidase Detection Kit; Pierce Biotechnology, Inc.) and developed on film. The band intensity of target protein levels relative to the housekeeping protein, β-actin, was quantified using the scanning densitometer and Quantity One Software, version 4.4.1 (Bio-Rad Laboratories).

**Table 2:** Primary antibodies used in western blotting analysis

**Name**	**Dilution**	**Catalog number**	**Company**
VEGF (Mouse monoclonal)	1:500	SC-7269	Santa Cruz Biotechnology, Inc., Santa Cruz, CA, USA
AQP-4 (Rabbit polyclonal)	1:2,500	SC-20812	Santa Cruz Biotechnology, Inc., Santa Cruz, CA, USA
GS (Mouse monoclonal)	1:2,000	MAB302	Millipore Corporation, Bioscience, Billerica, MA, USA
iNOS (Mouse monoclonal)	1:2,500	610329	BD Biosciences, Franklin Lakes, NJ, USA
eNOS (Mouse monoclonal)	1:500	610296	BD Biosciences, Franklin Lakes, NJ, USA
nNOS (Rabbit polyclonal)	1:500	610311	BD Biosciences, Franklin Lakes, NJ, USA
Nestin (Rabbit polyclonal)	1:500	SC-20978	Santa Cruz Biotechnology, Inc., Santa Cruz, CA, USA
β-Actin (Mouse monoclonal)	1:10,000	A 1978	Sigma, St Louis, MO, USA

### Glutamate assay

The glutamate concentration (mg/mL) in the retina protein supernatant of sham (*n*=3) and blast rats (*n* = 3 each at 24 h, 72 h, and 2 weeks post BH and BL exposure) were determined with a Glutamate BioAssay kit (US Biological, Swampscott, MA, USA; catalogue number G7114). The protein lysates were prepared as for western blotting and the bioassay measurements were performed according to the manufacturer’s protocol.

### Nitrite assay

The total amount of NO (μM) in the retina samples from sham rats (*n* = 4) and blast exposure rats (*n* = 4 each at 24 h, 72 h, and 2 weeks post BH and BL exposure) were assessed by the Griess reaction, using a colorimetric assay kit (US Biological, catalogue number N2577-01). The protein lysates were prepared as for western blotting and the NO^2-^ measurements were performed according to the manufacturer’s protocol.

### Double immunofluorescence

Sham rats (*n* = 4) and blast exposure rats (*n* = 4 each at 24 h, 72 h, and 2 weeks post BH and BL exposure) were used for immunofluorescence studies. At designated time-points after blast exposure, the rats were anesthetized with ketamine (75 mg/kg) and xylazine (10 mg/kg) administered intraperitoneally and then perfused transcardially with saline, followed by 4% paraformaldehyde in 0.1 M phosphate-buffered saline (PBS). The eyes were removed, post-fixed for 24 h in the same fixative and then dehydrated, embedded in paraffin, and sectioned sagittally into 4-μm-thick slices prior to immunofluorescence labeling. Briefly, after hydration, the tissue sections from different time points were rinsed in PBS, quenched for 10 min in methanol containing 3% H_2_O_2_, and incubated for 15 min in blocking solution (PBS containing 2% goat serum, 0.2% milk, and 0.1% Triton X-100), followed by incubation overnight in primary antibodies (Table 3). After incubation, FITC-conjugated and Cy3-conjugated secondary antibodies were added. Images representing at least one retina each from four rats at different time-points were captured under a confocal microscope (Olympus Fluoview TM1000; Olympus, Tokyo, Japan). Immunofluorescence labeling for the respective antibodies directed against the various cell types was consistent and reproducible across different rats. The isotypic control confirmed the specificity of all primary antibodies used (data not shown).

**Table 3:** Primary antibodies used in immunofluorescence studies

**Name**	**Dilution**	**Catalog number**	**Company**
AQP4 (Rabbit polyclonal)	1:250	SC-20812	Santa Cruz Biotechnology, Inc., Santa Cruz, CA, USA
VEGF (Rabbit polyclonal)	1:100	222-P	Thermo Scientific, Fremont, CA, USA
GFAP (Mouse monoclonal)	1:500	MAB360	Millipore Corporation, Bioscience, Billerica, Massachusetts, USA
GS (Mouse monoclonal)	1:2,000	MAB302	Millipore Corporation, Bioscience, Billerica, MA, USA
Nestin (Rabbit polyclonal)	1:500	SC-20978	Santa Cruz Biotechnology, Inc., Santa Cruz, CA, USA
Lectin (*Lycopersicon esculentum*)	1:100	L-0401	Sigma, St Louis, MO, USA

### Detection of apoptosis

For detection of cells undergoing apoptosis in the retina, paraffin sections as for double immunofluorescence labeling derived from sham rats (*n* = 3) and blast exposure rats (*n* = 3, each at 72 h and 2 weeks post BH and BL exposure) were assessed using a terminal deoxynucleotidyl transferase dUTP nick end labeling (TUNEL) apoptosis detection kit (Millipore Corporation, Billerica, MA, USA). After deparaffinization, the paraffin sections were hydrated briefly and subsequently washed with PBS, permeabilized with 0.2% Triton-X100 in PBS at room temperature. The remaining steps were performed according to the manufacturer’s instructions. TUNEL-positive cells were enumerated by counting the labeled cells in eight randomly selected microscopic fields obtained from each specimen at 40× objective. The percentage of TUNEL-positive cells was calculated and averaged. In addition, caspase-3 (1:100 dilution in PBS, NeoMarkers, Fremont, CA, USA, catalogue number RB-1197-P1) immunolabeling was carried out to confirm the presence of apoptotic cells.

### Retinal thickness assay

**Figure 1:**
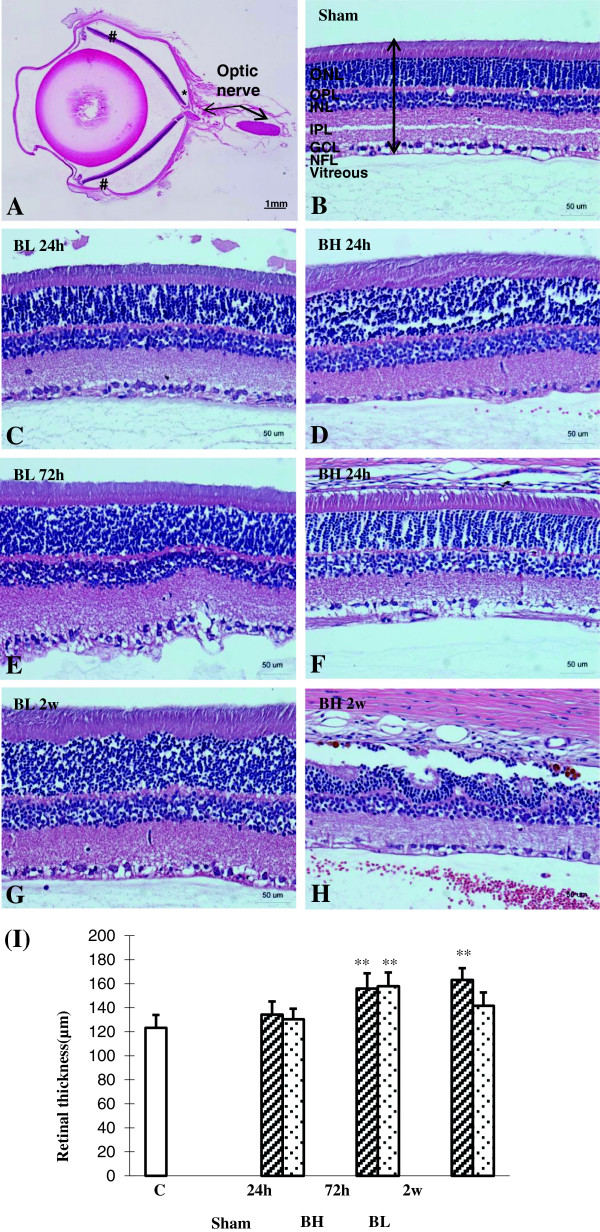
**Measurement of retinal thickness. (A)** Hematoxylin and eosin stain of sham rat eyeball for measurement of retinal thickness. At least five intact 20-μm sections were taken from the periphery (#), inclusive of the retina adjacent to the optic nerve head (*). Hematoxylin and eosin stain of a section of the **(B)** sham rat retina and blast exposure retina **(C-H)** for measurement of retinal thickness as identified by the arrow. Retinal layers: nerve fiber layer (NFL), ganglion cell layer (GCL), inner plexiform layer (IPL), inner nuclear layer (INL), outer plexiform layer (OPL), and outer nuclear layer (ONL). Scale bar= 1 mm **(A)** and 50 μm **(B - H)**. **(I)** Bar graphs show retinal thickness at different time-points after BH and BL exposure. Retinal thickness is significantly increased at 72 h and 2 weeks after BH exposure and 72 h after BL exposure when compared with that of the sham rats (mean±SD) (** *P* <0.01).

The sections for the quantitative assessment of the retinal thickness were from the same eyes used in the immunofluorescence labeling. Quantification of the thickness was performed in 4-μm-thick sagittal sections of the eyeball. Five intact sections, each separated by 20 μm, were evaluated for a span of 100 μm adjacent to the optic nerve head and along the periphery. In each selected section under 40× objective, the retinal thickness at three sites per section were measured using Image J (version 2.1.4.6 software; Image J, National Institutes of Health, Bethesda, MD, USA). The thickness was measured across the different retinal layers including the nerve fiber layer (NFL), ganglion cell layer (GCL), inner plexiform layer (IPL), inner nuclear layer (INL), outer plexiform layer (OPL), and outer nuclear layer (ONL) (Figure 1).

### Statistical analysis

For western blots and multi-analyte ELISArray, data are reported as mean ± SD. Data were analyzed using Mann–Whitney U test (SPSS version 15.0 software; SPSS, Chicago, IL, USA) to determine the statistical significance of differences between sham rats, rats subjected to BH exposure, and rats subjected to BL exposure. All experiments were conducted in triplicate from different tissue samples. Significance is accepted as *P* value <0.05 and is denoted by the asterisks (*P <0.05; **P <0.01).

## Results

### Increased retinal thickness and lesions after blast

By H&E staining, BL and BH retina were observed to have increased lesions as evidenced by increased spacing and disorganization of retinal layers in a severity-dependent manner, being most obvious at 72 h and 2 weeks after blast (Figure 1B-H). At 24 h after BL and BH exposures, the retinal thickness as measured from tissue sections was comparable to sham. However, it increased significantly at 72 h and 2 weeks after BL and BH exposures compared to the corresponding sham (*P* <0.01) (Figure 1I).

### Elevated inflammatory molecules after blast exposure

The concentration levels of the following cytokines and chemokines were assayed in both the protein lysates of retina of sham and blast exposure groups using the Multi-Analyte ELISArray: IL-1α, IL-1β, IL-2, IL-4, IL-6, IL-10, IL-12, IL-13, IFN-γ, TNF-α, GM-CSF, and RANTES. In the retinal tissue, there was no significant increase in all cytokines compared to sham at BH 24 h and BL 24 h, At 72 h. L-1α, IL-1β, IL-2, IL-10, IL-12, IL-13, IFN-γ, TNF-α, GM-CSF, and RANTES levels were increased significantly after BH compared to sham. At 2 weeks, all cytokines were significantly increased after BH compared to sham while IL-1β, IL-2, IL-6, IL-10, IL-12, IFN-γ, TNF-α, GM-CSF, and RANTES levels increased significantly after BL compared to sham. In addition, IL-1α, IL-12, and RANTES levels after BH exposure were significantly higher compared to BL exposure at 2 weeks.

**Figure 2:**
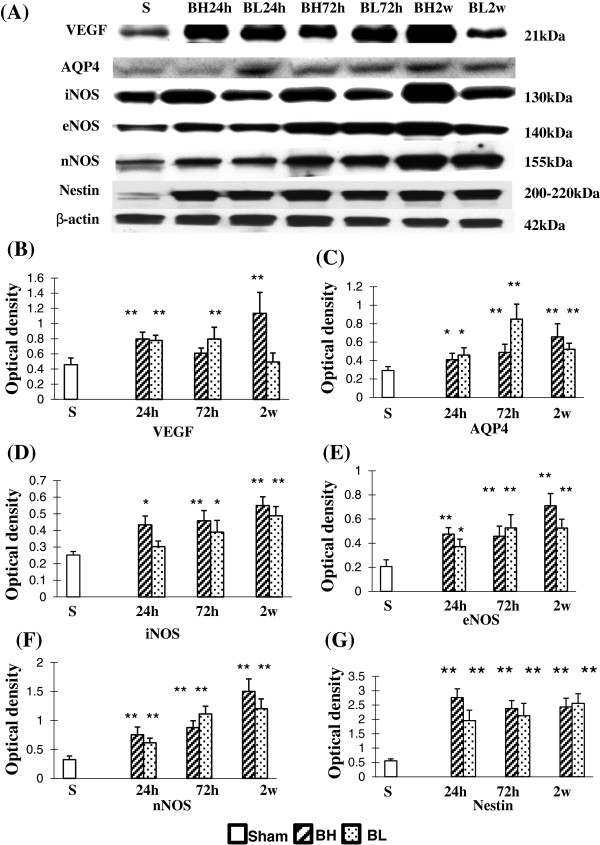
**Western blot analysis of VEGF, AQP4, iNOS, eNOS, nNOS, and nestin protein expression levels in the retina.** Sham rats (lane 1); rats subjected to BH exposure and sacrificed at 24 h (lane 2), 72 h (lane 4), and 2 weeks (lane 6); and rats subjected to BL exposure and sacrificed at 24 h (lane 3), 72 h (lane 5), and 2 weeks (lane 7). **(A)** VEGF (21 kDa), AQP4 (34 kDa), iNOS (130 kDa), eNOS (140 kDa), nNOS (155 kDa), nestin (200–220 kDa), and β-actin (42 kDa) immunoreactive bands. Bar graphs representing optical density (mean±SD) of **(B)** VEGF, **(C)** AQP4, **(D)** iNOS, **(E)** eNOS, **(F)** nNOS, and **(G)** nestin normalized to β-actin for sham, 24 h, 72 h, and 2 weeks after BH and BL (* *P* <0.05; **P <0.01). **(B)** VEGF shows an acute increase at 24 h and 72 h after BH and BL exposure when compared with that of the sham rats with recovery at 2 weeks after BL exposure when compared with the sham and at 2 weeks after BH exposure. **(C)** AQP4 shows a steady increase at 24 h, 72 h, and 2 weeks after BH and BL exposure when compared with that of the sham rats. **(D)** iNOS shows a significant increase at 24 h, 72 h, and 2 weeks after BH and BL exposure when compared with that of the sham rats. **(E)** eNOS and **(F)** nNOS show increase at 24 h, 72 h, and 2 weeks after BH and BL exposure when compared with that of the sham rats with decrease at 2 weeks after BL exposure when compared with the sham and at 2 weeks after BH exposure. **(G)** Nestin shows an acute and significant increase at 24 h, 72 h, and 2 weeks after BH and BL exposure when compared with that of the sham rats.

**Figure 3:**
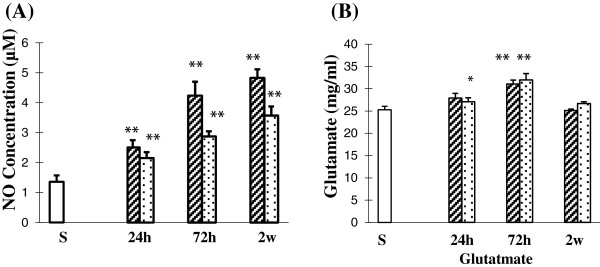
**Glutamate and NO levels (A-B) in retina of sham, BH, and BL rats. (A)** NO concentration is significantly increased in the retina at 24 h, 72 h, and 2 weeks after BH and BL exposure when compared with that of the sham rats; NO concentration is significantly increased in the retina at 72 h and 2 weeks after BH exposure when compared with that of 24 h after BH exposure; NO concentration is significantly increased in the retina at 2 weeks after BL exposure when compared with that of 24 h after BL exposure; NO concentration is significantly increased in the retina at 72 h and 2 weeks when compared with that of 72 h and 2 weeks after BL exposure, respectively. **(B)** Glutamate concentration is significantly increased in the retina at 24 h and 72 h after BH and BL exposure when compared with that of the sham rats with recovery at 2 weeks after BH and BL exposure; glutamate concentration is significantly increased in the retina at 72 h after BH and BL exposure when compared with that of 24 h and 2 weeks, respectively, after BH and BL exposure. (mean±SD) (* *P* <0.05; **P <0.01).

Besides cytokines and chemokines, VEGF, AQP4, GS, NO, NOS, and nestin have been reported to play important roles in inflammation in various tissues under pathological conditions, including the retina [17,19,23,25,26]. NO is a unique gaseous molecule that may act as a chemical messenger involved in vasodilation and neuroinflammation. *In vivo*, NO is produced and released from three different isoforms of NOS: iNOS, eNOS, and nNOS. By western analysis, iNOS-immunoreactive bands, with a molecular weight of approximately 130 kDa, showed a significant increase in optical density at 24 h after BH and BL exposure (*P* <0.05), 72 h after BH and BL exposure (*P* <0.01), and 2 weeks after BH and BL exposure (*P* <0.01) in comparison to the sham levels (Figure 2A, D). iNOS expression showed an increasing trend from 24 h to 2 weeks post blast for both BH and BL exposures. eNOS- and nNOS-immunoreactive bands, with a molecular weight of approximately 130 and 140 kDa, respectively, increased significantly in optical density at 24 h after BH and BL exposure (eNOS: *P* <0.01 and *P* <0.05; nNOS: *P* <0.05 and *P* <0.01), 72 h after BH and BL exposure (eNOS: *P* <0.01 and *P* <0.01; nNOS: *P* <0.01 and *P* <0.01) and 2 weeks after BH and BL exposure (eNOS: *P* <0.01 and *P* <0.01; nNOS: *P* <0.01 and *P* <0.01) compared with the sham levels (Figure 2A, E, F). NO levels in the retinal samples as determined by nitrite levels were significantly increased (*P* <0.01) in the retina by about +84%, +211%, and +255% at 24 h, 72 h, and 2 weeks, respectively, after BH exposure compared with sham. In BL group, nitrite levels were significantly increased (*P* <0.01) by about +58%, +112%, and +162% at 24 h, 72 h, and 2 weeks, respectively, compared with the sham (Figure 3A). At 72 h and 2 weeks nitrite levels were significantly higher in BH compared with BL exposure (Figure 3A) by about +69% and +92% (*P* <0.01).

**Figure 4:**
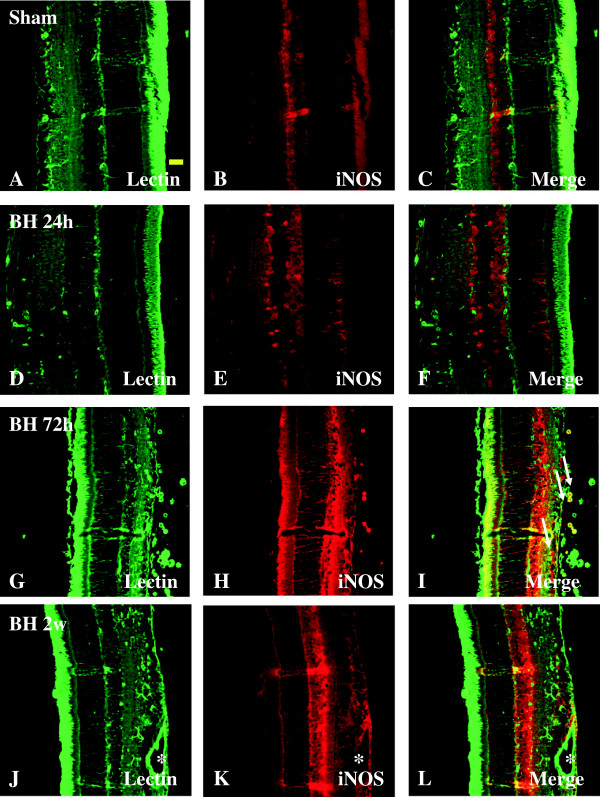
**Expression and distribution of iNOS (red) and lectin (green) in the retina of sham (A-C) and BH rats (D-L).** Lectin is localized mainly in the blood vessels (asterisk) and microglial cells (single arrow). In sham retina, a moderate iNOS labeling is detected in GCL, INL, and ONL **(B)**. Following blast exposure **(D-L)**, iNOS expression is strongly intensified when compared with the sham, especially in lectin-labeled microglial cells (single arrow), Müller cells (bold arrow), hyalocytes (asterisk), and the pigment cell layer. Scale bar=20 μm **(A-L)**.

**Figure 5:**
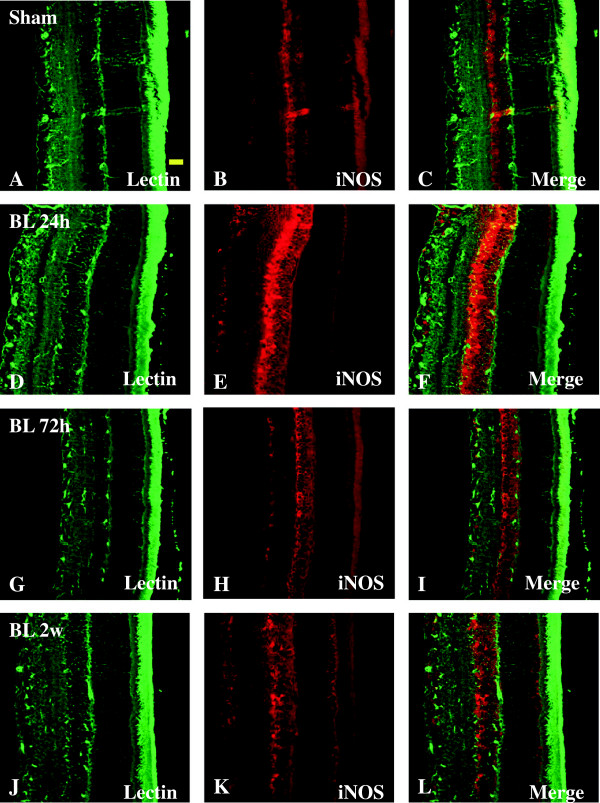
**Expression and distribution of iNOS (red) and lectin (green) in the retina of sham (A-C) and BL rats (D-L).** Lectin is localized mainly in the blood vessels (asterisk) and microglial cells (single arrow). In sham retina, a moderate iNOS labeling is detected in GCL, INL, and ONL **(B)**. Following blast exposure **(D-L)**, iNOS expression is strongly intensified when compared with the sham, especially in iNOS expression is strongly intensified when compared with the sham, especially in lectin-labeled microglial cells (single arrow), Müller cells (bold arrow), hyalocytes (asterisk), and the pigment cell layer. Scale bar=20 μm **(A-L)**.

Double-labeling was carried out using lectin for microglia expressing iNOS. In the sham rats, moderate iNOS labeling was present in the GCl, INL, and ONL but absent from lectin-labeled microglia in different layers of the retina (Figure 4B). At 24 h, 72 h, and 2 weeks following BH exposure, iNOS was induced and upregulated in in microglia, and remarkably also the Muller cells and processes and hyalocytes (in vitreous body), as well as the pigment cell layer as evidenced by double-labeling for ramified microglia and blood vessels (Figure 4A-L). A similar feature was observed in the retina of rats subjected to BL exposure (Figure 5A-L).

### Increased expression of edema-regulating molecules after blast

#### Expression of AQP4 and GFAP

**Figure 6:**
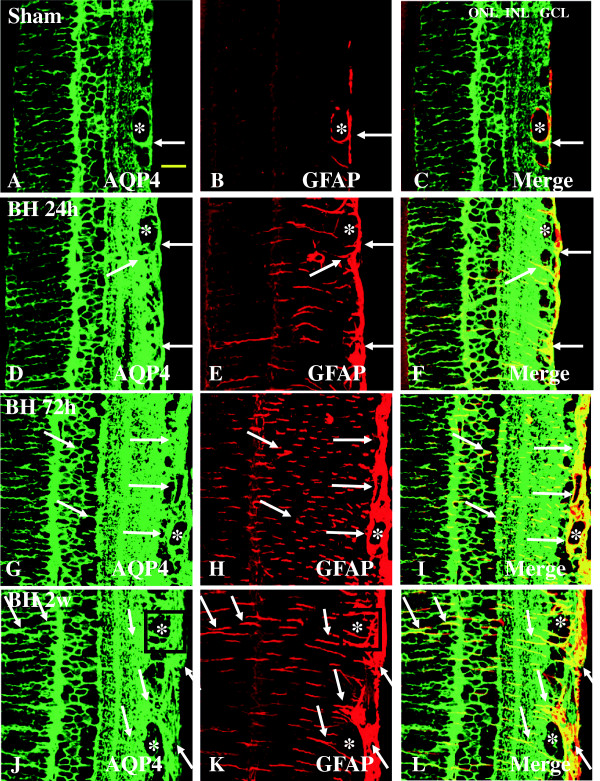
**Expression and distribution of AQP4 (green) and GFAP (red) in the retina of sham (A-C) and BH rats (D-L).** In the sham retina, AQP4 expression is localized in the blood vessels (asterisk), GFAP-labeled astrocytes (single arrow) **(A-C)**. Following blast exposure, AQP4 expression is markedly increased in various retinal layers at different time points (24 h, 72 h, and 2 weeks, **D-L**). Intense AQP4 immunoreactivity is localized in GFAP-labeled astrocytes and blood vessels. It is also markedly enhanced in the Müller cell process in parallel arrays **(D-L)**. AQ4 immunoreactivity remains intense in the blood vessels (asterisk) at different time points. Scale bar=20 μm **(A-L)**.

**Figure 7:**
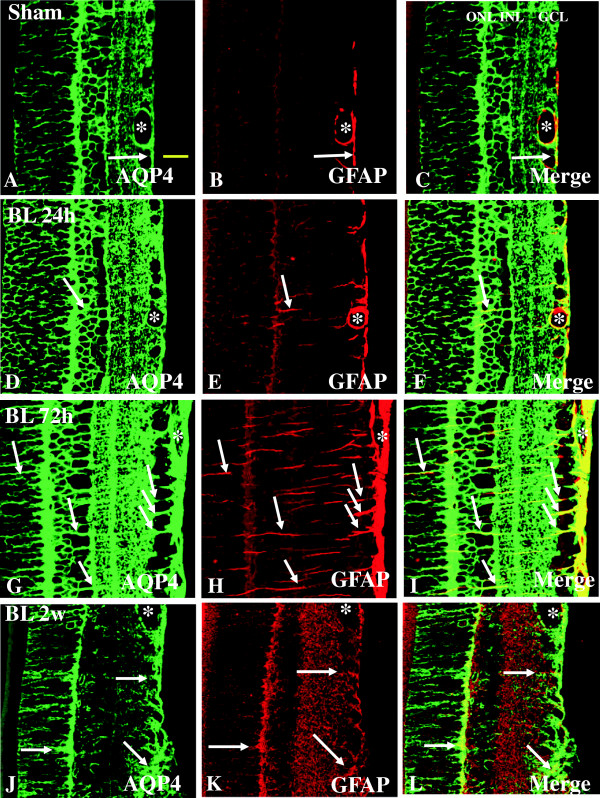
**Expression and distribution of AQP4 (green) and GFAP (red) in the retina of sham (A-C) and BL rats (D-L).** In the sham retina, AQP4 expression is localized in the blood vessels (asterisk), Müller cells, and GFAP-labeled astrocytes (single arrow) **(A-C)**. Following blast exposure, AQP4 expression is markedly increased in various retinal layers at different time points (24 and 72 h, **D-I**). Intense AQP4 immunoreactivity is localized in GFAP-labeled astrocytes and blood vessels. It is also markedly enhanced in the Müller cell process in parallel arrays **(D-I)**. At 2 weeks, AQP4 immunoreactivity is reduced to levels almost comparable to that of the sham **(J-L)**. AQ4 immunoreactivity remains intense in the blood vessels (asterisk) at all time-points. Scale bar=20 μm **(A-L)**.

AQP4 is a main water channel which is highly concentrated in the Müller cells and perivascular membranes of astrocytes in the retina. It has been suggested to be critically involved in the pathogenesis of retina edema under various injuries [13,17-19]. In the sham rats, moderate AQP4 labeling was detected in different retinal layers including the NFL, GCL, IPL, INL, OPL, and ONL (Figure 6A). In rats subjected to BH exposure, AQP4 expression was conspicuously increased in Müller cells whose processes appeared to extend across into the outer retina (Figure 6D, G). AQP4 immunofluorescence, however, was attenuated at 2 weeks and was comparable to that of the sham (Figure 6J). Robust AQP4 expression was observed at 72 h after BH exposure when compared with other time-points (Figure 6G). Glial fibrillary acidic protein (GFAP)-positive astrocytes and vascular profiles were coincident with AQP4 immunofluorescence in the sham rats and rats subjected to BH exposure (Figure 6A-L). Double labeling with anti-AQP4 and anti-GFAP confirmed that the AQP4-positive cells were astrocytes. In rats subjected to BL exposure, a similar immunostaining pattern was observed in the retina (Figure 7A-L). By western analysis, AQP4 was detected as a major band at approximately 34 kDa, and showed a significant increase in optical density at 24 h after BH and BL exposure (*P* <0.05), 72 h after BH and BL exposure (*P* <0.01), and 2 weeks after BH and BL exposure (*P* <0.01) in comparison with the sham levels (Figure 2A, C). The AQP4 protein expression level appeared to decline at 2 weeks after BL exposure but remained elevated above sham levels. After BH exposure, AQP4 expression showed an increasing trend from 24 h to 2 weeks post blast.

#### Expression of VEGF and GFAP

**Figure 8:**
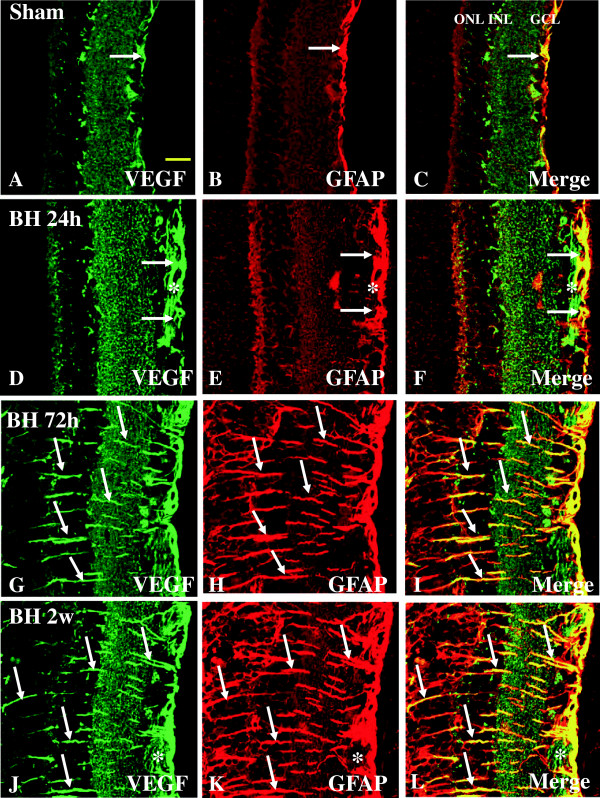
**Expression and distribution of VEGF (green) and GFAP (red) in the retina of sham (A-C) and BH rats (D-L).** In sham retina, a moderate VEGF labeling is detected in different retinal layers including the NFL, GCL, and IPL **(A)**. In the GCL, weak VEGF immunofluorescence is co-localized in GFAP-labeled astrocytes (single arrow, **A-C**). Following blast exposure, VEGF expression is evidently increased in various retinal layers at different time-points **(D-L)** notably at 72 h and 2 weeks **(G, J)**. Following blast exposure, VEGF immunoreactivity is localized in GFAP-labeled astrocytes (single arrow) and blood vessels (asterisk). Scale bar=20 μm **(A-L)**.

**Figure 9:**
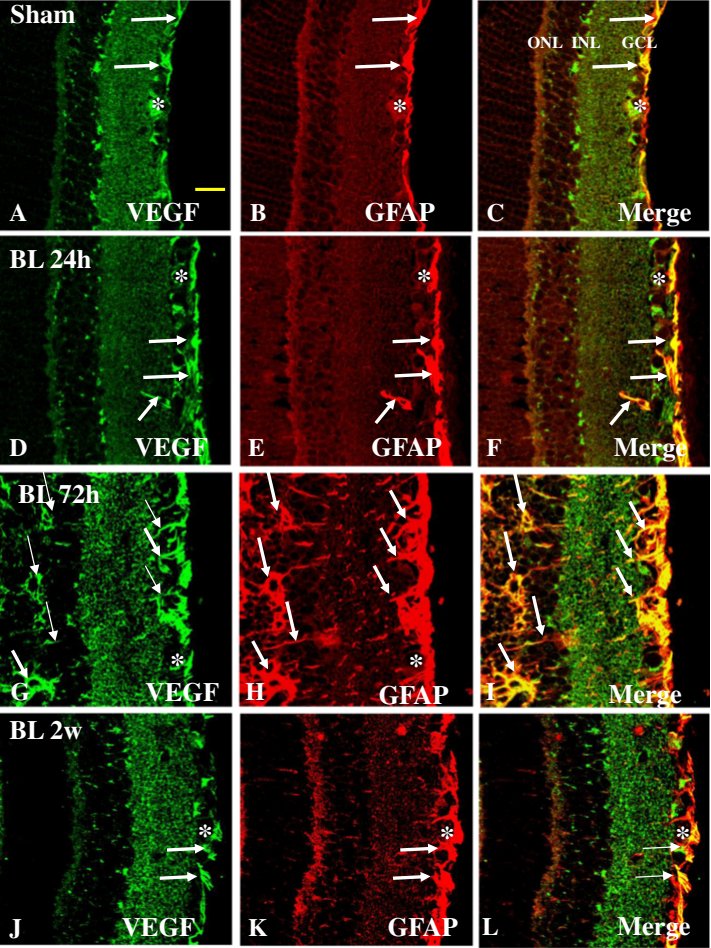
**Expression and distribution of VEGF (green) and GFAP (red) in the retina of sham (A-C) and BL rats (D-L).** In sham retina, a moderate VEGF labeling is detected in different retinal layers including the NFL, GCL, and IPL **(A)**. In the GCL, weak VEGF immunofluorescence is co-localized in GFAP-labeled astrocytes (single arrow, **A-C**). Following blast exposure, VEGF expression is evidently increased in various retinal layers at different time-points **(D-L)** being most intense at 72 h **(G)**. Following blast exposure, VEGF immunoreactivity is localized in GFAP-labeled astrocytes (single arrow) and blood vessels (asterisk). VEGF labeling in the soma of Müller cells appears to be more evident when compared with their processes **(I)**. Scale bar=20 μm **(A-L)**.

VEGF has been reported to stimulate vasodilation and leakage of water and large molecular weight proteins from blood vessels, resulting in edema [14]. In the sham rats, astrocytes confined to the NFL and GCL emitted GFAP immunofluorescence (Figure 8B). Co-localization of VEGF labeling with GFAP was evident, and was detected in the GCL only (Figure 8C). In rats subjected to BH exposure and killed at various time-points, the astrocytes had long extending and hypertrophic processes that were intensely stained for GFAP (Figure 8E, H, K). Müller cell processes in the palisade spanned the different retinal layers (Figure 8H, K); such a configuration was especially pronounced at 2 weeks post blast. Enhanced VEGF expression was most conspicuous at 2 weeks (Figure 8J) after BH exposure when compared with other time-points (Figure 8D, G). Except for some vascular profiles, GFAP-positive astrocytes in NFL were completely co-localized with VEGF immunofluorescence (Figure 8F, I, L). Both VEGF and GFAP immunoreactivity in astrocytes were associated with the blood vessels and was markedly enhanced after BH exposure (Figure 8G, J). A similar feature was observed in the retina of rats subjected to BL exposure (Figure 9A-I). By western analysis, VEGF-immunoreactive band of approximately 21 kDa showed a significant increase at 24 h after BH and BL exposure (*P* <0.01), 72 h after BL exposure (*P* <0.01) and 2 weeks after BH exposure (*P* <0.01) when compared with that of the sham rats. VEGF protein expression level was significantly decreased at 2 weeks after BL exposure compared to 2 weeks BH exposure (*P* <0.01) (Figure 2A, B).

#### Expression of nestin and GFAP

**Figure 10:**
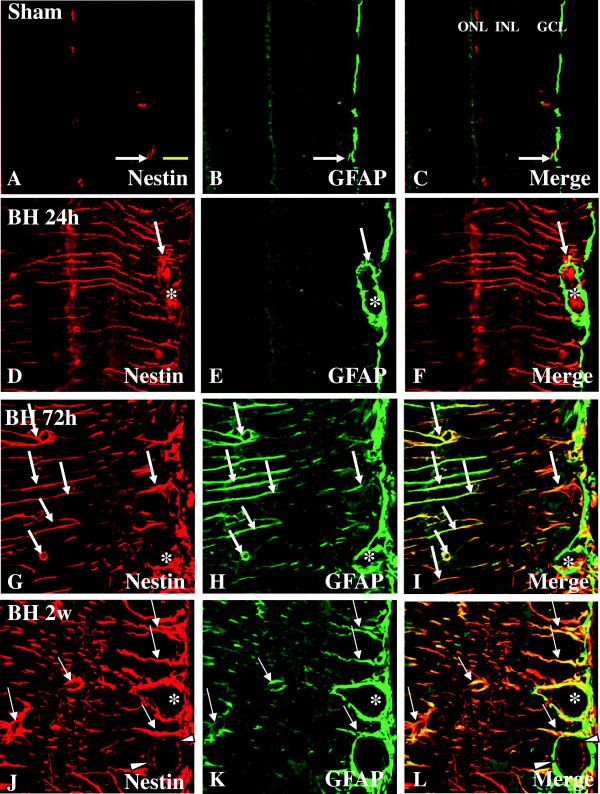
**Expression and distribution of nestin (red) and GFAP (green) in the retina of sham (A-C) and BH rats (D-L).** In sham retina, a weak nestin labeling is detected in GCL and IPL **(A)**. In the GCL, weak nestin immunofluorescence is co-localized in GFAP-labeled astrocytes (single arrow, **A-C**). Following blast exposure, nestin expression is evidently increased in various retinal layers at different time-points **(D-L)** notably at 72 h and 2 weeks **(G, J)**. Following blast exposure, nestin immunoreactivity is localized in GFAP-labeled astrocytes (single arrow) associated with blood vessels (asterisk). Nestin labeling in the vascular endothelial cells is evident (arrow head, **J-L**). Scale bar=20 μm **(A-L)**.

**Figure 11:**
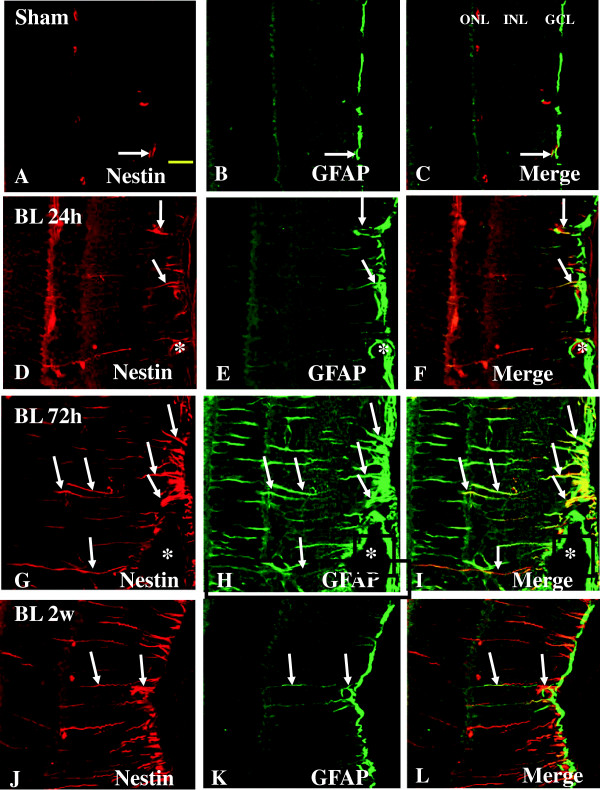
**Expression and distribution of nestin (red) and GFAP (green) in the retina of sham (A-C) and BL rats (D-L).** In sham retina, a weak nestin labeling is detected in GCL and IPL **(A)**. In the GCL, weak nestin immunofluorescence is co-localized in GFAP-labeled astrocytes (single arrow, **A-C**). Following blast exposure, nestin expression is evidently augmented in various retinal layers at different time-points **(D-L)** notably at 72 h and 2 weeks **(G, J)**. Following blast exposure, nestin immunoreactivity is localized in GFAP-labeled astrocytes (single arrow) and blood vessels (asterisk). Scale bar=20 μm **(A-L)**.

Müller cells, which are the principal retinal glial cells, are metabolically coupled to photoreceptors. The induced expression of nestin along with the increased expression of GFAP in Müller glial cells may reflect a metabolic change of the cells in response to the degenerative changes of their neighboring neurons whose functions are closely linked. In the sham rats, nestin labeling was barely detected in the in GCL and IPL (Figure 10A). Co-localization of GFAP and nestin appeared as punctate immunofluorescence that was detected in the GCL only (Figure 10C). At various time-points after BH exposure, Müller cells whose processes traversing different retinal layers were not only hypertrophied, but also exhibited intense nestin immunofluorescence (Figure 8H, K). Such a feature was especially pronounced at 2 weeks (Figure 10J) when compared with other time-points (Figure 10D, G). A noteworthy feature was the induced expression of nestin but not GFAP in Müller cells/processes at 24 h BH. In the NFL, GFAP-positive astrocytes and blood vessels were partly co-localized with nestin immunofluorescence (Figure 10I, L). Nestin immunoreactivity was markedly enhanced in astrocytes, after BH exposure (Figure 10G, J). Likewise, it was augmented in the vascular endothelial cells (Figure 10J-L). Double-labeling with anti-nestin and anti-GFAP revealed that the nestin-positive cells in NFL were astrocytes (Figure 10I, L). A similar feature was observed in the retina of rats subjected to BL exposure (Figure 11A-I). By western analysis, Nestin was detected as a major band at approximately 200–220 kDa, and showed a significant increase in optical density at 24 h after BH and BL exposure (*P* <0.01), 72 h after BH and BL exposure (*P* <0.01), and 2 weeks after BH and BL exposure (*P* <0.01) in comparison with the sham levels (Figure 2A, G). Nestin protein expression level was significantly lower at 24 h after BL exposure compared to the corresponding sham at 24 h after BH exposure (*P* <0.05) (Figure 2A, G).

### Changes in glutamate metabolism and cell death after blast

**Figure 12:**
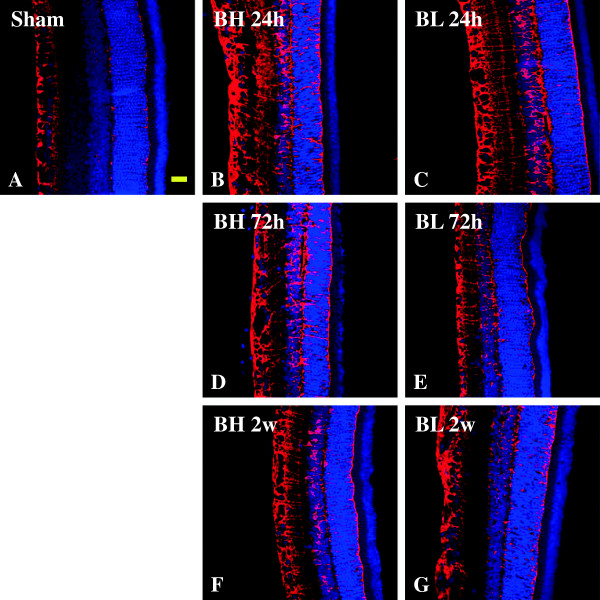
**Expression and distribution of GS (red) and DAPI (blue) in the retina of sham (A) and BH (B,D,F) and BL (C,E,G) rats.** In the sham rats **(A)**, GS immunofluorescence is moderately expressed in the OPL. Following blast exposure **(D-L)**, GS expression is strongly enhanced when compared with the sham in a severity-dependent manner for which immunofluorescence is most robust at 24 h. Scale bar=20 μm **(A-G)**.

The function of Müller cells is in the maintenance of retinal water/potassium and glutamate homeostasis which makes them important players in photoreceptor survival [19]. Glutamate levels in the retinal samples were significantly increased beginning at 24h after BH and BL exposure (*P* <0.05) peaking at 72 h after BH and BL exposure (*P* <0.01) in comparison with the sham levels (Figure 3B). Glutamate levels, however, showed a significant decrease at 2 weeks after BH and BL exposure (*P* <0.01) compared to 72 h and restored to levels comparable to that of the sham. By immunohistochemistry, at 24 h to 2 weeks after BH and BL exposure, GS immunofluorescence was remarkably enhanced in Müller cells being most robust at 24 h after injury (Figure 12A-G).

**Figure 13:**
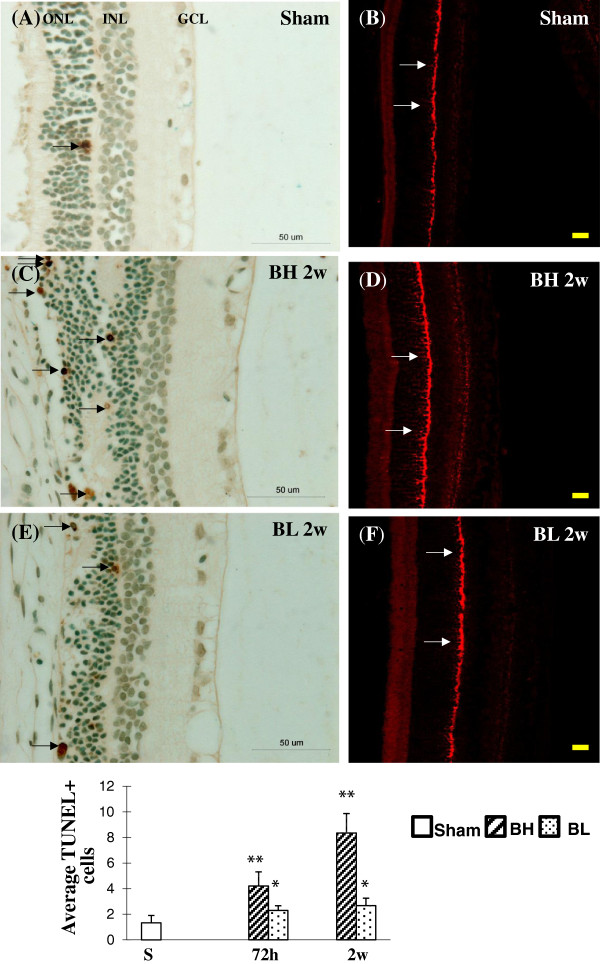
**Distribution of TUNEL and caspase-3 immunoreactive cells in the retina of sham rats (A, B), BH 2 w (C, D) and BL 2 w rats (E, F).** Blast exposure, especially BH **(C, E)**, increases the incidence of apoptotic cells (black arrows) in the ONL compared to sham animals **(A)**. Scale bar=50 μm **(A, C, E)**. Similarly, blast exposure, especially BH **(D, F)**, results in an increase in caspase-3 immunofluorescence (red) in the neurons in or between the outer and inner granular cell bipolar layers compared to sham animals **(A)**. Scale bar=20 μm **(B, D, F)**. **(G)** Bar graphs show increase in apoptotic cells at 72 h and 2 weeks after BH and BL exposure when compared with that of the sham rats. The frequency of apoptotic cells is significantly increased in the retina at 2 weeks after BH exposure when compared with that of 72 h after BH exposure and 72 h and 2 w after BL exposure, respectively (mean±SD) (* *P* <0.05; **P <0.01).

BH and BL exposure-induced apoptosis of cells was observed in the ONL using TUNEL and caspase-3 immunostaining (Figure 13A-F). Interestingly, incidence of apoptosis of cells in the GCL and INL region in the retina was less obvious (Figure 13A,C,E). Similarly, caspase-3 was observed to be upregulated in the neurons in or between the outer and inner granular cell bipolar layers (Figure 13B,D,F). No caspase-3 immunopositive cells were found in the retinal ganglion and photoreceptor cell layers. The number of retinal cells undergoing apoptosis as evidenced by TUNEL labeling was markedly increased (*P* <0.05, *P* <0.01) in rats subjected to BH exposure at 72 h, and 2 weeks after BH and BL exposure when compared with sham rats (Figures 13G). Apoptotic cells were significantly increased in numbers (*P* <0.01) in the retina at 2 weeks after BH exposure compared with that of 72 h after BH exposure and 72 h and 2 weeks after BL exposure (Figure 13G).

## Discussion

The effects of primary blast injury on the brain have been well-investigated [27]. As far as can be ascertained, the effects of primary blast injury on the retina have not been reported. In view of this lack of information, we have chosen VEGF, AQP4, GS, iNOS, glutamate, and nestin as the main parameters for investigation as these factors have been reported to be closely linked to inflammation, apoptosis and edema in the brain, spinal cord, and lung after primary blast exposure [9-11,13,16,17,19,21,25,26].

It has been reported that a single exposure to BOP results in neuronal and glial cell damage, along with compromised vascular permeability and inflammation [28]. Combined with bioinformatics analysis and interpretation of primary microarray data, these methods would generate a new level of understanding about cerebral edema, inflammation, and neuronal death cascades [29]. The results of the serum cytokine measurements following blast exposure signified a delayed inflammatory response [30]. One study reported that systemic IL-1, IL-10, and VEGF receptor Neuropilin-2 (NRP-2) were elevated predominantly after primary blast exposure in the serum of rats subjected to primary blast [16]. The increase in cytokines in the retinal tissue was most evident at 72 h after BH exposure and 2 weeks after BH and BL exposure, notably at 2 weeks in rats subjected to BH exposure suggesting a robust blast intensity-dependent inflammatory response in the retina in the present experimental model after a primary blast injury and that inflammation may be elevated and sustained. Of the cytokines investigated, the quantum of increase was most substantial for IL-12, TNF-α, and RANTES.

We have reported enhanced tissue concentration of VEGF in the hypoxic retina [13]. Here we show increased VEGF immunofluorescence in the blood vessels and astrocytes as verified by their co-localization with GFAP. This suggests that the blood retinal barrier (BRB) may be compromised leading to retinal edema, with corresponding increases in retinal thickness. Besides VEGF, the present study has shown a significant increase in NO production in the retinal tissue after BH and BL exposure. iNOS was not only induced in microglia, but remarkably also the Müller cells, hyalocytes (in vitreous body), as well as the retinal pigment cell layer suggesting the contribution of NO from many different retinal cellular sources. We reported increased NO production in the retina in response to smoke inhalation [13]. NO has also been implicated in the regulation of vascular permeability [20,21]. We have previously reported evidence of combustion smoke inhalation induced increased NO and alteration in the retinal tissue of adult rats, and administration of iNOS inhibitor prevented the development of leakage in the retinas of rats subjected to smoke exposure [13]. In separate studies, astrocytes may have contributed to excess production of NO as these cells are also known to express iNOS under stressful conditions [31,32]. Expression of iNOS, eNOS, and nNOS protein levels was concomitantly and progressively increased at 24 h, 72 h, and 2 weeks after BH and BL exposure. It is suggested that NO derived from the various NOS isoforms can contribute to increased retinal blood vessel permeability and result in edema in primary blast exposure conditions. The concurrent and enhanced protein expression of VEGF, iNOS, eNOS, and nNOS at different time points in the retina after BH and/or BL exposure support their inter-relationship [33,34]. It would also appear therefore that multiple factors such as enhanced production of VEGF, NO, and NOS may be jointly responsible for causing pathophysiology to the retina following primary blast injury.

The present results have shown that AQP4 protein expression was increased in the retina after primary blast exposure. By double immunofluorescence labeling, increased expression of AQP4 was detected in the retina primarily in the blood vessels, astrocytes, and Müller cells. This supports the view that the astrocytes and Müller cells are involved in the transport of water from the blood vessels to the retina. It also suggests a close relationship between BRB function and the sham of water flux by astrocytes and Müller cells as evidenced by the hypertrophic processes of astrocytes and Müller cells in the primary-exposed retina. In addition, AQP subunits have been reported to play differential roles in various astroglial responses (including astroglial swelling and astroglial loss) in the chronic epileptic hippocampus [35].

The present results have shown that BH and BL exposure-induced apoptosis of cells were observed in the ONL. A similar upregulation in caspase-3 was observed in the neurons in or between the outer and inner granular cell bipolar layers. It was reported that there was increased apoptosis of astrocytes and oligodendrocytes in the brain of *Macaca fascicularis* following primary blast exposure [27]. A similar result was reported by [9]. In the rabbit model of explosive blast injury to the spinal cord, co-existent apoptotic and necrotic changes in cells were reported [10]. No caspase-3 positive cells were observed in the retinal ganglion and photoreceptor cell layers. This may be due to the unique mechanism of blast-induced injury differing from the other types of retinal injuries reported in the literature. Although the nature of the apoptotic cells cannot be determined with certainty, this finding suggests that primary exposure would affect the visual function. Remarkably, visual impairment was observed in some rats at 2 weeks after BH exposure (unpublished observation), consistent with the above-mentioned experimental results. Furthermore, the high VEGF expression may contribute to increased apoptosis of retinal cells.

It has been reported that NO plays an important role in neuronal injury [22,36]. The present results have also shown the upregulation of NO and NOS in the retina of rats subjected to primary blast exposure. These suggest that the acute production of NO through the increased expression of the various NOS can lead to subsequent apoptosis affecting the ONL whose cells may be more vulnerable to blast injury-induced cell death.

In a mouse model of retinal degeneration, photoreceptor degeneration was associated with excessive free glutamate levels and with an upregulation of glutamate turnover. This suggests that excessive glutamate levels contribute to photoreceptor degeneration [24]. The present results have also shown that glutamate levels in the retinal samples were significantly increased at 24 and 72 h after BH and BL exposure in comparison with the sham levels. In addition, the incidence of BH and BL exposure-induced apoptotic cells was increased in the ONL. A recent cDNA microarray study showed significant changes in the expression of glutamate receptors in the midbrain region along with significant changes in multiple genes involved in inflammatory pathways in various regions of the brain suffering from blast-induced traumatic injury [37] and suggests that the same inflammatory mechanism may be ongoing in blast-exposed retina.

The glutamate levels in the retinal samples were concomitantly increased at 24 h after BH and BL exposure and 72 h after BH and BL exposure in comparison with the sham levels while GS immunoexpression level was enhanced in Müller cells after blast. This suggests that glutamate homeostasis is disrupted after blast exposure be it BL or BH. Furthermore, the glutamate-induced GS expression suggests the presence of a protective mechanism against glutamate-induced excitotoxicity and may explain for the low incidence of apoptotic cells despite upreglation of caspase-3 immunofluorescence in the ONL [13,38].

The induced expression of nestin along with the increased expression of GFAP in Müller glial cells may reflect a metabolic change of the cells in response to the degenerative changes of their neighboring neurons whose functions are closely linked. Hence, nestin as well as GFAP is a useful biomarker for retinal injury [39]. In a recent study of amyloid-induced inflammatory responses in the rat retina, induced inflammatory responses were characterized by increases in markers for microglia and astrocytes (ionized calcium-binding adaptor molecule 1 (Iba-1), GFAP and nestin) [19]. A major finding in this study was the drastic increase in nestin expression in Müller cells and their processes at various time-points after BH and BL exposure. It is unequivocal that primary blast injury induces Müller cell response as manifested not only by an increase in GFAP and nestin expression but also by the hypertrophy of their processes. This along with increased GS suggests that Müller cells may play a protective mechanism in regulating glutamate homeostasis. The induced expression of nestin also suggests the ‘stemness’ of Müller cells in response to adverse conditions and it is unclear how this phenomenon contributes to blast retinal pathophysiology.

The present study has shown that the changes in the retina as reflected by the increased expression of various molecules involved in modulation of inflammation, edema, and apoptosis was acute in onset after a single primary blast exposure. This reinforces the notion of blast-induced retinal injury and highlights the need for protective eyewear that can effectively mitigate against such injury for potential victims and well as soldiers in the battlefield. The present results have shown that BH exposure results in more drastic changes than BL exposure in terms of expression levels of inflammatory cytokines and chemokines, VEGF, NOS, NO, GS, and nestin. In light of this, we conjecture that the degree of primary blast-induced injury in the retina is proportional to the varying BOP exposure. These blast-induced retinal changes appeared to be sustained till 2 weeks after blast exposure. This suggests that there may be developing injury pathophysiology and that the blast effects on the retina could be long-lasting. In addition, retinal ONL cells seem to be vulnerable to blast injury given the upregulation of various parameters tested post blast. Based on the parameters tested, potential therapeutics such as VEGF antibodies and anti-inflammatories may be investigated. The chronic presence of blast retinal injury as depicted in our study should be taken into consideration when determining the window for therapeutic intervention as well as for diagnosis and prognosis for blast retinal injury. Finally, the role of astrocytes and Müller cells in blast retinal injury pathophysiology remains to be elucidated. Astrocytes may serve as a double-edged sword in promoting edema by upregulating AQP4 expression yet may serve as a protective mechanism in a supporting role. Separately, the involvement of Müller cells remains unclear especially with regards to the upregulation of nestin after blast exposure which points towards a possibility of neurogenesis from these modified ‘Müller stem cells’. The function of nestin-expressing Müller cells in relation to blast retinal injury requires further investigation.

## Conclusion

We report here that a single primary blast exposure upregulates the expression or production of VEGF, AQP4, glutamate, iNOS, eNOS, nNOS, NO, and nestin in the adult rat retina, accompanied by the rise in pro- and anti-inflammatory cytokines and chemokines. Remarkably, an increase in incidence of apoptotic cells was also observed in the ONL of the retina after primary blast exposure which seem to be most vulnerable to blast injury as opposed to other forms of retinal injury Blast exposure can also increase BRB permeability and contribute to tissue edema as shown by the increased thickness of the affected lesioned retina. It also elicited drastic response in astrocytes and Müller cells. It is suggested that these changes are collectively involved in the above pathological injuries of the retina in primary blast exposure. Finally, BL and BH induced BOP-dependent injuries being more severe with higher BOP exposure. These retinal pathological changes as manifested by the increased expression of different molecules involved in inflammation, edema, and apoptosis after primary blast injury appear to sustain for at least 2 weeks.

## Abbreviations

AQP4: Aquaporin-4; BA: Body armor; BH: Blast high; BL: Blast low; BOP: Blast overpressure; BRB: Blood-retinal-barrier; eNOS: endothelial NOS; GCL: Ganglion cell layer; GS: Glutamine synthethase; Iba-1: Ionized calcium-binding adaptor molecule 1; iNOS: inducible NOS; IPL: Inner plexiform layer; INL: Inner nucler layer; NFL: Nerve fibre layer; NO: Nitric oxide; NOS: Nitric oxide synthase; nNOS: neuronal NOS; ONL: Outer nuclear layer; OPL: Outer plexiform layer; PBS: Phosphate-buffered saline; PETN: Pentaerythritol tetranitrate; TBI: Traumatic brain injury; TBS: Tris-buffered saline; TNT: 2,4,6-trinitrotoluene; TUNEL: Terminal deoxynucleotidyl transferase dUTP nick end labeling; VEGF: Vascular-endothelial growth factor.

## Competing interests

The authors declare that they have no competing interests.

## Authors’ contributions

EAL and JL designed the project, contributed to the analysis of data, and finalization of the manuscript. YYZ conducted all experiments and prepared the first draft of the manuscript. EMK conducted experiments and participated in discussion and analysis of data as well as editing the manuscript. KCN designed and conducted the blast experiment. MHT and LLY carried out the immunohistochemical staining. All authors have read and approved the final version of the manuscript.
